# Equitable and effective clinical guidance development and dissemination: trauma aims to lead the way

**DOI:** 10.1136/tsaco-2023-001338

**Published:** 2024-12-20

**Authors:** Lacey N LaGrone, Deborah M Stein, Danielle J Wilson, Eileen M Bulger, Ashley Farley, Andrés M Rubiano, Maria Michaels, Meghan B Lane-Fall, Michael A Person, Vanessa P Ho, Linda Reinhart, Elliott R Haut

**Affiliations:** 1 Trauma Acute Care Surgery, Medical Center of the Rockies, Loveland, Colorado, USA; 2 UCHealth, Loveland, Colorado, USA; 3 Department of Surgery, University of Maryland School of Medicine, Baltimore, Maryland, USA; 4 Department of Surgery, Medical College of Wisconsin, Milwaukee, Wisconsin, USA; 5 Department of Surgery, University of Washington, Seattle, Washington, USA; 6 Bill and Melinda Gates Foundation, Seattle, Washington, USA; 7 Neuroscience Institute, Universidad El Bosque, Bogota, Colombia; 8 Meditech Foundation, Cali, Colombia; 9 Centers for Disease Control and Prevention (CDC), Atlanta, Georgia, USA; 10 Perelman School of Medicine at the University of Pennsylvania, Philadelphia, Pennsylvania, USA; 11 Department of Surgery, University of South Dakota, Sioux Falls, South Dakota, USA; 12 Department of Surgery, Case Western Reserve University, Cleveland, Ohio, USA; 13 Department of Population and Quantitative Health Sciences, Case Western Reserve University, Cleveland, Ohio, USA; 14 Grand View Health, Sellersville, Pennsylvania, USA; 15 Department of Surgery, Johns Hopkins University School of Medicine, Baltimore, Maryland, USA

**Keywords:** guidelines, Clinical, injury

## Abstract

Thirty-four per cent of deaths among Americans aged 1–46 are due to injury, and many of these deaths could be prevented if all hospitals performed as well as the highest-performing hospitals. The Institute of Medicine and the National Academies of Science, Engineering and Medicine have called for learning health systems, with emphasis on clinical practice guidelines (CPGs) as a means of limiting preventable deaths. Reduction in mortality has been demonstrated when evidence-based trauma CPGs are adhered to; however, guidelines are variably updated, redundant, absent, inaccessible, or perceived as irrelevant. Ultimately, these barriers result in poor guideline implementation and preventable patient deaths. This multidisciplinary group of injury providers, clinical guidance developers and end users, public health and health policy experts and implementation scientists propose key areas for consideration in the definition of an ideal future state for clinical guidance development and dissemination. Suggestions include (1): professional societies collaborate rather than compete for guideline development.(2) Design primary clinical research for implementation, and where relevant, with guideline development in mind.(3) Select clinical topics for guideline development through systematic prioritization, with an emphasis on patient-centered outcomes.(4) Develop guideline authorship groups with a focus on transparency, equity of opportunity and diversity of representation.(5) Establish a plan for regular review and updating and provide the date the guideline was last updated for transparency.(6) Integrate options for adapting the guideline to local resources and needs at the time of development.(7) Make guidelines available on a platform that allows for open feedback and utilization tracking.(8) Improve discoverability of guidelines.(9) Optimize user-experience with a focus on inclusion of bedside-ready, mobile-friendly infographics, tables or algorithms when feasible.(10) Use open access and open licenses.(11) Disseminate clinical guidance via comprehensive and equitable communication channels. Guidelines are key to improve patient outcomes. The proposed focus to ensure trauma guidelines are equitably and effectively developed and disseminated globally.

## Lay summary

 Many deaths from injuries can be prevented if all hospitals worked as well as the best hospitals. Clinical guidance gives healthcare workers advice on how to treat patients and can improve patient outcomes, but it is only followed half the time. Some problems with trauma clinical guidance are that it is not always updated, it is hard to find, there can be different advice on the same topic, and sometimes no advice at all. We believe improving clinical guidance can help take better care of injured patients and save lives. We suggest the following: (1) professional groups should work together to write guidance. (2) Make sure clinical research is designed to be used in practice. (3) Ensure guidance covers topics that are important to patients. (4) Include diverse individuals in writing guidance. (5) Create a plan to update guidance regularly. (6) Provide advice for healthcare providers who have less access to supplies or specialists. (7) Use an electronic platform to postguidance and track how it is used and how it can be improved. (8) Make guidance easier to find. (9) Make guidance easier for healthcare workers to use when seeing patients. (10) Ensure guidance is free to use. (11) Make sure guidance is shared in a way that no one is left out.

### Injury burden and the current state of trauma guidelines

There is unacceptable variability within the US health system for trauma care, which consists of medical interventions for injured patients provided by a multidisciplinary team of surgeons, emergency physicians, nurses, prehospital personnel and others.^1^ In 2020, an estimated 5.3 million years of potential life were lost due to injury, more than any other pathology in the USA.^2 3^ Additionally, 34% of deaths among Americans aged 1–46 are due to injury, many of which could be prevented if all hospitals performed as well as the highest-performing hospitals.^1 3 4^ These metrics are mirrored globally, with an estimated 34%–38% of injury deaths being potentially preventable when outcomes are adjusted based on the highest-performing centers.^5^ Additionally, poor outcomes, including preventable deaths, are disproportionately experienced among persons who are uninsured, non-white, woman, or reside in rural or low-income urban settings.^6^,^9^ A meta-analysis revealed uninsured trauma patients have more than double the odds of death compared with those who are insured and, when controlling for other socioeconomic barriers, ethnicity remains independently associated with the quality of care received.^6 9^ Geographic location also impacts patient outcomes, with delays in transport time increasing mortality.^7 8^ Several factors contribute to these findings, such as trauma center distribution and implicit bias; however, improving the quality, equity and consistency of care delivered is key and can be achieved, in part, through clinical practice guidelines (CPGs).^8 10^

Adherence to clinical guidance, a series of evidence-based recommendations to optimize patient care, is associated with decreased trauma patient mortality.^11^,^14^ In a multi-institutional study, major deviations from guidelines applicable to injured patients were associated with a threefold increase in mortality at 30 and 90 days.^12^ Additionally, the Brain Trauma Foundation found an associated 50% reduction in mortality when their severe head injury guidelines were implemented.^14^ Guidance implementation also reduces healthcare costs. While cost-savings for trauma care have not been assessed, a global review of guidance utilization showed cost reduction in 10 of 11 studies, ranging from 6%–57%.^15^ Despite these findings, trauma guidelines are only utilized in 50%–60% of patients to whom they apply.^12 13^

**Figure 1:**
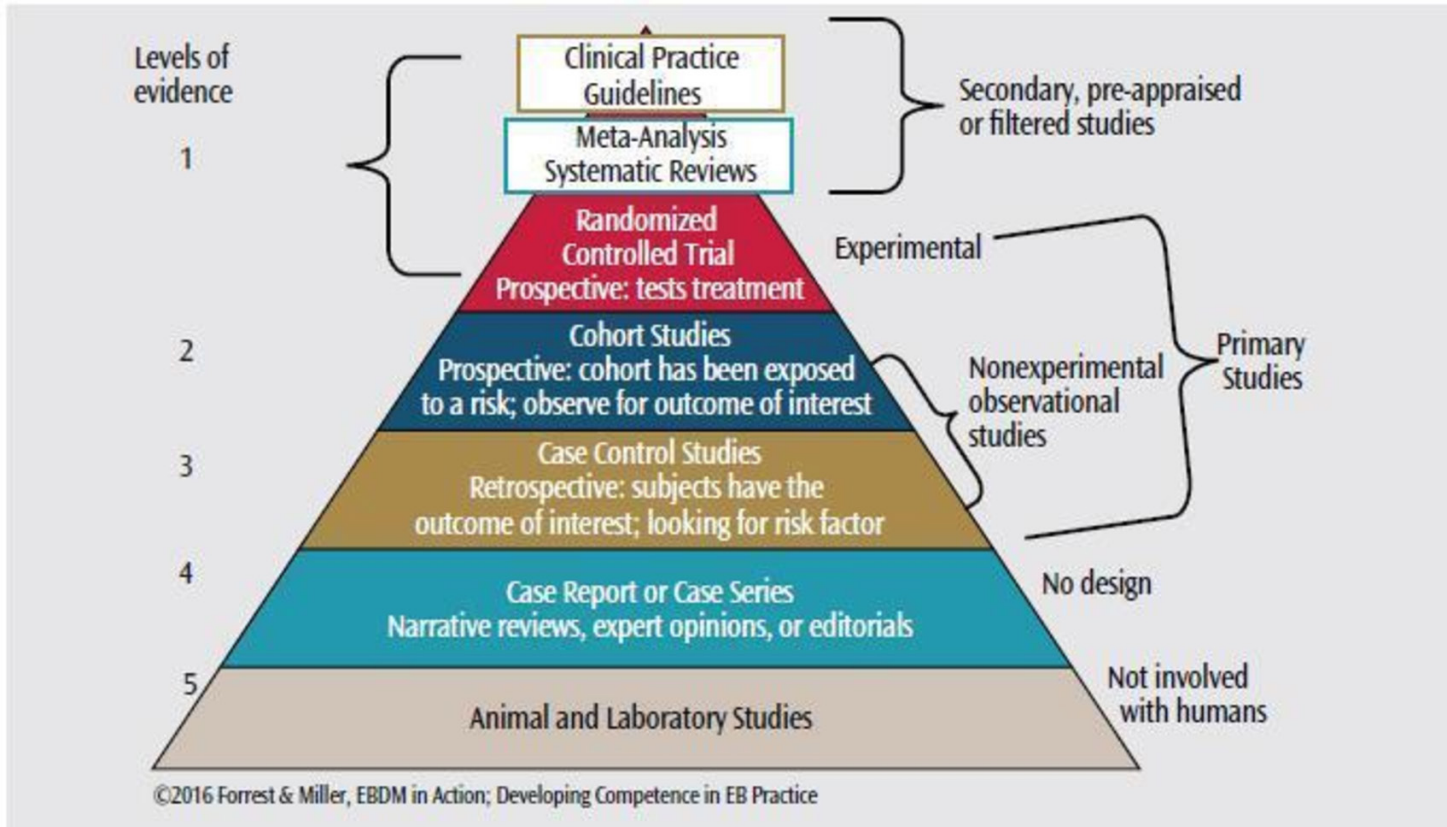
The evidence-based pyramid for clinical medicine.

Several reasons exist for poor implementation, including many guidelines becoming outdated just 3 years after publication and applicability only to specific clinical situations or contexts, making them irrelevant for many others.^11 14^ The absence of active distribution, or dissemination, leaves providers unaware of existing guidelines, and those who are aware may lack access to journals that publish guidelines, such as those in rural or global practice.^16 17^ When guidelines do reach providers, the lengthy publications are not user-friendly to enact at the bedside.^18^ Finally, redundant CPGs from multiple organizations often reach different conclusions from the same evidence, leaving providers uncertain which recommendations to enact.^11 14^ While some suggest CPGs should be at the top of the evidence-based medicine pyramid (figure 1), it is essential to address these deficiencies in development, accessibility and implementation.

Furthermore, while cumulative data regarding diversity of guideline authorship are lacking, there is consistent evidence that persons from the global majority (eg, non-white people) are disproportionately under-represented among surgeons, particularly among full professors of surgery.^19 20^ Acknowledging that diverse teams have been shown to result in better innovation and scientific productivity, the current lack of professional diversity further hampers the effectiveness of existing guideline development methods.^21^

### The path forward for clinical guidance

In response to the unacceptable variability in outcomes across disciplines, coupled with the increasing healthcare costs, the Institute of Medicine (IOM) proposed ‘learning health systems’.^2^ The IOM states, ‘although unprecedented levels of information are available, patients and clinicians often lack access to guidance that is relevant, timely and useful for the circumstances at hand’. Learning health systems target factors controllable by the provider, institution and/or system, but currently do not address social determinants of health and resource constraints that also contribute to variable outcomes. The 2016 National Academies of Science, Engineering and Medicine report titled, ‘A National Trauma Care System, Integrating Military and Civilian Trauma Systems to Achieve Zero Preventable Deaths After Injury’ identifies guidelines as an integral component of learning health systems.^1 22^

Public and patient involvement (PPI) is central to learning health systems and associated with improved patient experience, quality of life and health outcomes.^2^ Due to these associations, guideline development standards describe PPI as vital to ensuring the views and preferences of those impacted by guidelines are incorporated.^23^ Despite this recommendation, only 8% of guideline developers have utilized PPI, and 20% of guidelines have a lay-friendly version, in part due to lack of a detailed methodology on how to incorporate patients into established processes.^23^ This represents a crucial gap that must be remedied in future guideline development.

Furthermore, inspired by the CDC’s ‘Adapting Guidelines for the Digital Age’ initiative, one can envision a future where trauma clinical research and guidelines are equitably ‘designed for implementation’ from their inception.^24^ This includes integrating guidelines into clinical workflows. Fast Healthcare Interoperability Resources (FHIR) is a standard required for health IT (Information Technology) certification by the Office of the National Coordinator for Health IT (ONC) in the USA (eg, electronic health records (EHRs)). FHIR Clinical Guideline, more commonly known as ‘CPG-on-FHIR’, is an international standard to represent guidelines in computable form to allow implementation into any EHR, for example, as shareable and interoperable clinical decision support (CDS). Research is needed to quantify the impact of implementing FHIR-based computable guidelines, especially in surgery. However, literature shows improved decision-making, diagnosis and reduction in extraneous testing when CDS is utilized.^18^ Artificial intelligence and more advanced CDS may eventually address some of the gaps in health information access.^25^ However, for patients currently experiencing preventable poor outcomes, a more organized and collaborative approach to guideline development and implementation is warranted.

Professional trauma organizations offer different approaches to evidence-based medicine, including the Eastern Association for the Surgery of Trauma (EAST) ‘guidelines’, the Western Trauma Association ‘algorithms’, the American College of Surgery Committee on Trauma, Trauma Quality Improvement Program (ACS-COT TQIP) ‘best practices’, the American Association for the Surgery of Trauma (AAST) ‘tools’, and most recently, the AAST-COT joint ‘protocols’.^26^ In response to disjointedness in competitive grant funding acquisition and coordination of multi-institutional trials, the Coalition for National Trauma Research (CNTR) was formed to improve US trauma research coordination.^27^ CNTR’s mission supports implementation of best practice trauma care as a core value and this national-level trauma organization inspired us to consider the options regarding clinical guidance.^17^

Additionally, US hospitals routinely undergo external review for verification, and professional trauma societies have expectations for patient allocation, partnering with local governments to enforce shared public health and policy initiatives.^28^ With this strong foundation of collaboration, standardization and implementation, trauma is well positioned to lead progressive efforts to improve the evidence base and ensure equitable dissemination and implementation. Development of transparent, best practices for guideline development, will not only benefit the trauma community but also create a standard that may be utilized across specialties.

### Key suggestions to improve trauma clinical guidance

This multidisciplinary group of injury providers, professional society leadership, clinical guidance developers and end-users, public health and health policy experts and implementation scientists propose 11 key areas for consideration (table 1). Recommendations result from consensus-building techniques utilizing a problem-solving-focused approach that was anchored in defining an ideal future state for clinical guidance development and dissemination.^29^ A single-text approach was employed, in which a draft of initial perspectives was serially edited through remote meetings and written communication in preparation for an Agency for Healthcare Research and Quality (AHRQ) grant application entitled, *Design for Implementation: The Future of Trauma Research and Clinical Guidance*, which was ultimately awarded. All initial suggestions were maintained and are presented in an order that flows from conceptualization to implementation of guidelines.

**Table 1:** Current challenges and proposed solutions in trauma clinical guidance

Challenges in trauma CPG development and implementation	Proposed solutions
Guideline redundancy	Professional society collaboration on guideline development
Deficit in translation of research from bench-to-bedside	Design primary clinical research for implementation
Gaps in guidelines	Systematic prioritization of guideline development topics with patient and public involvement
Disparities in authorship	Focus on transparency, equity of opportunity, and diversity in representation of authorship team
Outdated recommendations	Establish timeline for regular review and update of CPGs and include date of last update
Lack of relevance in disparate clinical contexts	Integrate adaptations based on local resources during guideline development
Lack of feedback on relevance and applicability of individual CPGs	Develop a platform that allows feedback and utilization tracking
Difficult identification of existing guidelines	Improve discoverability through user feedback
Diverse formats limit bed-side readiness	Optimize user experience with infographics, tables, or algorithms and include lay versions for patient interpretation
Cost-prohibitive access	Publish in open access and open license formats for physician and patient use
Poor dissemination	Employ comprehensive and equitable communication channels for dissemination

CPG, clinical practice guideline

1. **Professional societies collaborate rather than compete for guideline development**. Collaboration could limit redundancy, reduce conflicting guidance, improve the perceived authority of existing guidelines and enhance discoverability.^11 14 30^ Professional societies and other consensus bodies should work together to leverage resources and make our collective library more comprehensive, our author lists more inclusive, and provider work less cumbersome.
2. **Design primary clinical research for implementation, and where relevant, with guideline development in mind**. Research should use core outcome sets to facilitate interpretation and comparison of results in development of evidence-based guidelines.^31^ Pragmatic trials and hybrid effectiveness-implementation trials offer key means of bridging the ‘know-do’ gap.^32^ Hybrid trials, which concurrently study the impact of an intervention and strategies to promote its uptake, permit a focus on real-world relevance while gaining understanding of implementation mechanics. This shortens the time from knowledge generation to integration into practice and improves the representativeness of the patient populations studied. Plain language summaries and/or key points could make the information more understandable to a broader audience. These efforts may aid to leap-frog from primary literature to practice, while integrating into other efforts within the knowledge transformation ecosystem, all without requiring the creation of new content (ie, new guidelines).
3. **Select clinical topics for guideline development through systematic prioritization, emphasizing patient-centered outcomes**. While guideline developers consistently cite ‘burden of disease’ as the primary reason for topic selection, a recent systematic review suggests erratic application of other meaningful motivators drive selection (eg, practice variation, equity relevance, economic impact, feasibility of practice change).^33^ Recognizing outcome gaps in previously identified patient priority areas or directly engaging patients and patient advocate organizations in prioritization would achieve this goal. When involved, patients are often approached after data synthesis, but their voice is imperative at each stage to ensure that efforts address their needs and preferences.^34^
4. **Develop guideline authorship groups focusing on transparency, equity of opportunity and diversity of representation**. Societies that publish guidelines within the US have a diversity, equity and inclusion (DEI) committee, subcommittee or representative. These resources can create processes that ensure guideline authorship represents the target audience in practice setting, gender, provider type and region. Pursuing ‘diversity in the diversity’ as well put by one such DEI lead can increase transparency, equity of opportunity and diversity of representation in the decision-making that affects the guideline recommendations. Additionally, several studies have found conflicts of interest permeate CPGs, limiting their objectivity and trustworthiness.^11^ Increasing authorship diversity and ensuring transparency are two ways to combat this issue.
5. **Establish a plan for regular review and revision and provide the date the guideline was last updated for transparency (as suggested by the IOM**).^35^ Clinical guidelines reflect a best effort to make informed recommendations given the current evidence. Nevertheless, that evidence is always growing. Timeliness is essential to ensure patients do not suffer unnecessarily because their provider did not have an up-to-date understanding of diagnostic and therapeutic options. Frameworks for living guidelines that undergo routine updates as new evidence emerges have been proposed, with a version history tracking the latest revision date.^36^
6. **Integrate options for adapting the guideline to local resources and needs at the time of development**. Rather than expending resources on a guideline which may only be narrowly relevant to certain resource settings, we recommend the inclusion of ‘if/then’ statements to make a guideline relevant to all providers, regardless of access to resources.^30^ The Colombian Consensus Guideline for Traumatic Brain Injury provides a ground-breaking example of this approach. The algorithm includes clinical branch points (eg, ‘Is the patient unresponsive?’) and resource branchpoints (eg, ‘is a nearby facility with neurosurgical services available?’).^37^ Furthermore, as guidelines become more adaptable and thus widely relevant, we recommend that language translation and context adaptation be considered at inception. An immediately feasible approach is to leverage existing professional society partnerships (eg, the AAST and the Panamerican Trauma Society) to create joint committees with an anticipated bidirectional exchange of expertise to develop guidelines implementable in multiple settings.
7. **Make guidelines available on a platform that allows for open feedback and utilization tracking**. Publishing guidelines through a dynamic channel would allow clinicians to provide feedback on CPG improvement target areas such as resource relevance, readability and perceived validity. This can be carried out via electronic platforms, like web-based or mobile applications, which enable providers to submit comments on specific guidance. The collected feedback can be collated, potentially using AI to identify key themes, and routinely reviewed by the guidance development team for necessary clarifications and updates. Reliable and transparent tracking of guideline downloads and/or access rates could then inform prioritization of guideline development, update, and even subsequent primary research.^18^ This would be more meaningful than citation tracking as the primary goal of a guideline is clinician access and application, rather than use by other authors or scientists in subsequent work or, in some cases, refutation.
8. **Improve discoverability of guidelines**. While some practitioners are facile in navigating society websites or identifying ideal search terms for online medical library queries, the variability of current guideline dissemination, even when open access, is a barrier to bedside application. Understanding how clinicians access information when they have clinical uncertainty will allow for a more user-centered design of dissemination strategies. These may include a central repository, standard taxonomies, voluntary inclusion on listservs that update clinicians when new guidelines are published, integration into the EHR, or others.^36 38^
9. **Optimize user experience with a focus on inclusion of bedside-ready, mobile-friendly infographics, tables or algorithms when feasible**. Currently, there are a myriad of formats, and while useful for research, they can be difficult to apply clinically. Consideration of formats that integrate into provider workflows is imperative, such as quick reference guides with algorithms.^35^ Improvements may require resource allocation for user-experience experts who are active in other industries but historically under-represented in clinical medicine. Additionally, lay-friendly access and readability, which is currently lacking, is essential for patient-centered care.^23^
10. **Disseminate clinical guidance via comprehensive and equitable communication channels**. Diverse communication channels should be used for active dissemination of information with an emphasis on groups that are historically under-reached, such as rural, non-academic and non-physician providers. A communication workgroup is one mechanism by which diverse means of dissemination can be identified during guideline development to ensure recommendations are reaching the intended users.^18^
11. **Use open access and open licenses**. There are various publication models and costs with conducting, reviewing and publishing research.^39^ However, consensus recommendations that are intended for bedside clinical care ought to fall under the axiom ‘to share is human’, as quoted in a recent paper sponsored by the CDC and AHRQ.^38^ Even if ‘sharing’ comes at the cost of short-term revenue generation for publishing companies, open access, and open licenses provides the most visibility for guidelines. Ensuring CPGs are accessible outside of urban, academic, high-resource centers is essential for their utility. Additionally, the public, who have contributed to research through public funding and participation in research, have a right to access these publications.^16^

There are several examples of best practice, including the former AHRQ Guidelines Clearinghouse, Guidelines International Network repository, and EAST repository.^26 40^ A model program is the National Cancer Care Network, which has an expansive catalog of relevant, up-to-date guidelines, and goes a step further to include international adaptations and translations.^41^ Importantly, to facilitate the more sophisticated aims for ultimate guideline development (such as integration of algorithms into EHRs, training artificial intelligence, etc.), it is essential that ‘open’ means fully open—where the materials may be reused, remixed, or translated (eg, creative commons (CC) BY license), shareable, and ideally interoperable in repositories such as in AHRQ’s CDS Connect that is based on the international CPG-on-FHIR health IT standard.

1. **Disseminate clinical guidance via comprehensive and equitable communication channels**. Diverse communication channels should be used for active dissemination of information with an emphasis on groups that are historically under-reached, such as rural, non-academic and non-physician providers. A communication workgroup is one mechanism by which diverse means of dissemination can be identified during guideline development to ensure recommendations are reaching the intended users.^18^

### Barriers and future research

Implementing these key recommendations is not without challenges. Guidelines are frequently cited in the literature, driving guideline development organizations’ desire for authorship, thus bolstering their citation metrics. One potential mechanism for collaboration without complete integration is a collective registry where guidelines are registered at the time of inception, thus limiting redundancy. A registry leverages existing impetus while limiting inefficiencies as a first step. Additional changes in how guideline development organizations and journals credit publications in the citation metrics may be necessary to facilitate collaboration and address other emerging trends, such as living guidelines. Furthermore, guideline creation is a long process, requiring months to years.^35^ Adding additional steps to incorporate PPI, address multiple resource contexts, include user-friendly formats and provide regular updates is even more labor-intensive and could create further delays. However, assembly of diverse, multidisciplinary teams and adaptation of current processes will help achieve these goals in a streamlined approach.

There are many end-user groups and key stakeholders, including all levels of providers from pre-hospital personnel to physicians in diverse specialties and practice environments, patients and experts in guideline development and dissemination, whose perspectives must be included to develop sustainable improvements in trauma clinical guidance. Though not all groups are represented in our authorship currently, capturing the unique experiences and ideas of all stakeholders is imperative to ensuring the development of a system that meets the needs of all users. Future research on optimal CPG format and dissemination is imperative to ensure guidelines are applicable for all end-users, regardless of the clinical context.^35^ Integration of computable guidelines into EHRs, ensuring they reach the correct clinical scenario without undue alert fatigue, is one approach that requires additional efforts.^18^ Further research to optimize methodologies to incorporate PPI into guideline development is also necessary.^23^ Additionally, implementation barriers must continue to be uncovered to create change at a systems level and, lastly, key metrics in guideline utilization and patient outcomes must be identified to ensure meaningful and sustainable changes are taking place.^18^ The suggestions above strive to address these gaps in knowledge by engineering in feedback loops, allowing for continued improvement.

The authors acknowledge having published in journals that colleagues and even co-authors could not afford to access and participating in academic trends that result in retention of knowledge within existing hierarchies. This is a call to action by trauma providers, and their allies, for trauma providers to work towards a more ideal future for clinical guidance development and dissemination.
